# A Fast and Precise Tool for Multi-Layer Planar Coil Self-Inductance Calculation

**DOI:** 10.3390/s21144864

**Published:** 2021-07-16

**Authors:** Andreia Faria, Luís Marques, Carlos Ferreira, Filipe Alves, Jorge Cabral

**Affiliations:** 1 ALGORITMI Center, University of Minho, 4800-058 Guimarães, Portugal; jcabral@dei.uminho.pt; 2 Centre of Physics of Minho and Porto Universities, 4710-057 Braga, Portugal; lsam@fisica.uminho.pt; 3 PhD AESI Bosch Car Multimedia, 4705-820 Braga, Portugal; id6642@alunos.uminho.pt; 4 Integrated Micro and Nanotechnologies, International Iberian Nanotechnology Laboratory, 4715-330 Braga, Portugal; filipe.alves@inl.int; 5 CEiiA—Centro de Engenharia e Desenvolvimento, 4450-017 Matosinhos, Portugal

**Keywords:** planar coil, self-inductance, mutual-inductance, analytical tool, multi-layer inductance coil, finite element method

## Abstract

An open-source tool that allows for a fast and precise analytical calculation of multi-layer planar coils self-inductance, without any geometry limitation is proposed here. The process of designing and simulating planar coils to achieve reliable results is commonly limited on accuracy and or geometry, or are too time-consuming and expensive, thus a tool to speed up this design process is desired. The model is based on Grover equations, valid for any geometry. The validation of the tool was performed through the comparison with experimental measurements, Finite Element Model (FEM) simulations, and the main analytical methods usually used in literature, with errors registered to be below 2.5%, when compared to standard FEM simulations, and when compared to experimental measurements they are below 10% in the case of the 1-layer coils, and below 5% in the 2-layer coils (without taking into consideration the coil connectors). The proposed model offers a new approach to the calculation of the self-inductance of planar coils of several layers that combines precision, speed, independence of geometry, easy interaction, and no need for extra resources.

## 1. Introduction

Over the years, the growth of solutions based on technologies using planar coils has been perceptible [1,2]. Due to its fabrication and operating characteristics, planar coils offer solutions with a lower weight, better mechanical stability, and volume efficiency, when compared to solenoids, enabling its use in a wide range of applications [2,3]. Additionally, since planar coils can be printed on traditional circuit boards (PCB) or on flexible materials, a highly repeatable, predictable, and economically efficient production can thus be achieved, facilitating assembly and integration processes [4,5,6]. The combination of this production method with the high reliability of inductive based technologies, results in the creation of solutions that offer robustness, durability, good thermal behavior, flexible design, high replication capability, for applications in wet and harsh conditions, like the presence of oil and dust [7,8]. Due to its characteristics and functionalities, PCB based planar coils meet many of the requirements imposed by competitive industries, such as automotive, healthcare, robotics, electronics, from low-power to high-power applications [9,10,11]. In the automotive industry, planar coils have been widely used, for example in Electric Vehicles (EVs) [12], through the integration of wireless power transfer (WPT) systems. The evolution of these systems will contribute to the improvement of the dynamic wireless charging (DWC) process, mitigating some of the major barriers for EVs adoption related to the management of stored energy, battery capacity, charging time, and the high costs associated with it [12]. In the medical devices industry, planar coils have also been used to charge implantable devices [9,13]. This allows one not only to reduce the size of the implantable devices, since smaller batteries are needed, but also reduces the need for replacement surgeries and consequently the risk of patient infections or damage to organs or muscle tissues. Planar coils are also used in different sensing applications, due to their ability to satisfy the constraints related to the device’s size, the manufacturing costs, and harsh operating conditions. Currently, there are already in the market displacement and angular position sensors that integrate planar coils in their transduction mechanism, such as the eddy current effect or inductive coupling [2,14,15], like the magnet-free IPS2200 inductive position sensors produced by Renesas company [16].

Considering the strong interest of several industries in planar coil technology, and the huge competitiveness of the markets, the development cycle of new devices has to be shortened, to keep up with the fast pace of competitors [2]. The design and optimization of planar coils is a complex, costly and time consuming process, usually based on finite element modeling (FEM) methods and experimental measurements [17,18,19]. Fast, closed form analytical methods can also be found in the literature [1,8,20,21,22,23,24,25,26] for coil optimization. However, these methods are limited to the calculation of the self-inductance of regular coils with specific geometries.

In this work an analytical tool to calculate the self-inductance of generic multi-layer planar coils is proposed, combining precision, versatility, and speed. The main goal is to improve and simplify the design process of planar coils, with minimal computational resources, allowing the analysis of how different coils’ design parameters (such as the number of turns, the space between turns, the turns width, the number of segments per turn, and the inner and outer diameters) influence the coil’s inductance. The following sections present and validate the analytical model proposed. Section 2 shows the analytical expressions commonly used to calculate the self-inductance of planar coils, Section 3 details the proposed analytical calculation method of planar coils, while Section 4 validates the proposed model through the comparison with FEM simulations, and experimental measurements. In order to have a more complete validation, different coil geometries and dimensions were used and analysed. In Section 5, the main conclusions of the model validation are drawn and its effectiveness is discussed.

## 2. Review of Analytical Models

Unlike in the case of solenoids, the internal area of a planar coil is dependent on its outer diameter, the number of turns, the width of the wire, and the space between turns. Thus, this type of coils has the particularity that all turns have different lengths and, because of this, the calculation of the self-inductance of flat coils becomes more challenging. The auto-inductance of a coil is dependent on the mutual inductance between each current turn, the self-inductance of the coil’s wire, and the environment surrounding it. Having this in consideration, together with the particularities of planar coils previously mentioned, the complexity associated with the auto-inductance calculation for flat coils design is perceptible.

According to the literature [1,8,20,21,22,23,24,25,26,27,28,29], to define the design of a coil for an application, two types of methodologies stand out, the numerical methods, e.g., finite element method (FEM), and the approximation formulas. Although FEM simulation is the most accurate methodology, it requires expensive simulation programs, high computational resources, and the FEM model development itself is a meticulous and complex process. It also has the disadvantage that the simulation of the model can take from minutes to several hours or days, depending on the complexity of the model, the desired precision, and the number of cases and variables in simulation. Due to these aspects, this method may become more appropriate for design verification than for its development [7]. Thus, a methodology based on approximation formulas is ideal to overcome part of the disadvantages of FEM simulations, since it is no longer required to use high computational resources and dedicated software tools. This methodology consists of applying the chosen formula, replacing the model variables with the coil design parameters, and therefore it is much faster when compared to a full FEM simulation. However, this simplicity brings some limitations regarding the coil geometries that the approximation applies for, as well as the precision of the model results. Based on the literature [1,8,20,21,22,23,24,25,26,30], there are several approximation formulas for calculating the inductance of planar coils, some are application specific [18,30,31,32], or are only valid for one type of geometry such as the research works from Bryan, Terman, Olivei, Gleason, Dill and others [1,26,33,34], some remain valid for several regular geometries like the Data Fitted Monomial, the Current Sheet Approximation and the Modified Wheeler [1,24,26,35]. These last ones, due to their higher versatility and precision in the results, when compared to others proposed in the literature, are the ones most commonly used. These approximation formulas were developed by Mohan et al. [35] and validated for coils with an outside diameter ($d_{out}$) between 100–480 μm, an inside diameter ($d_{in}$) between 0.1–0.9 $d_{out}$, a wire width (*w*) between 2 μm and 0.3 $d_{out}$, a space between turns (*s*) from 2 μm to 3 $d_{out}$, and an inductance value between 0.5 and 100 nH.

**Figure 1:**
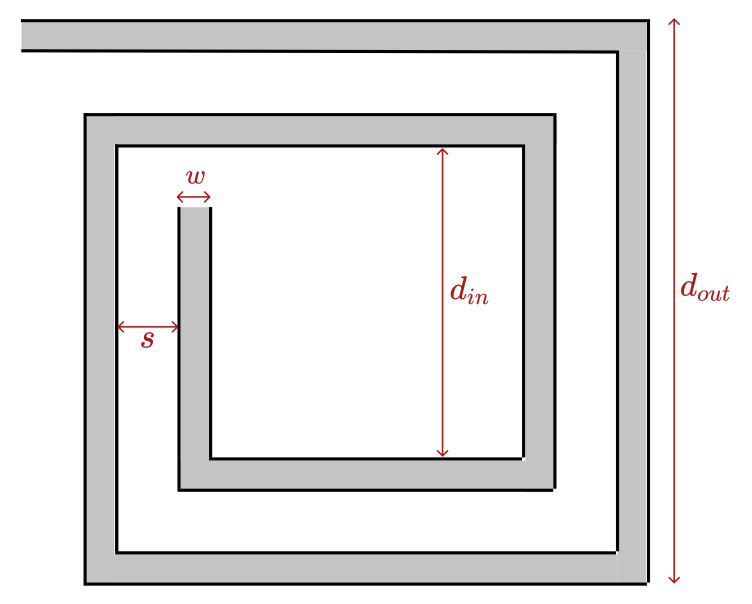
Square coil model for the approximation formulas.

The expression Data Fitted Monomial was developed using the authors’ database of inductors, athwart data-fitting techniques. For that reason, when the characteristics of the coil under analysis are different from those of the database, the error between the formula and the coil inductance can be significant. The coil geometries that can be used and the respective coefficients are presented in Table 1. The inductance formula relies on this set of geometry dependant coefficients and in the coil’s parameters to calculate the self inductance:

(1) $$L=\beta d_{out}^{\alpha_{1}}w^{\alpha_{2}}d_{avg}^{\alpha_{3}}N^{\alpha_{4}}s^{\alpha_{5}}$$

where: $d_{out}$ corresponds to the outer-diameter, *w* to the turn’s width, $d_{avg}$ to the average diameter ($d_{avg}=\frac{d_{out}+d_{in}}{2}$), $d_{in}$ to the inner-diameter, *N* to the number of turns, and *s* to the space between turns (visible in Figure 1).

The Current Sheet Approximation formula is based on the approximation of the sides of the coil to the current sheets of equal density using electromagnetic principles. In this formula, the calculation of the auto and mutual inductances between the coil wires is performed considering concepts of geometric mean distance (GMD), the arithmetic mean distance (AMD), and the arithmetic mean square distance (AMSD) [35]. Due to this approach, the greater the ratio between the space between turns and the track’s width, the larger the errors between this formula and the real coil inductance. Table 2 shows the coil layouts for which this approach is valid and the corresponding coefficients to be applied:

(2) $$L=\frac{\mu_{0}N^{2}d_{avg}c_{1}}{2}\left(\ln\left(\frac{c_{2}}{\rho }\right)+c_{3}\rho +c_{4}\rho \right)$$

where, $\mu_{0}$ is the vacuum permeability equal to $4\pi \times 10^{-7}\;N/A^{2}$, $d_{avg}$ is the average diameter, *N* the number of turns, and ρ is the fill ratio ($\rho =\frac{d_{out}-d_{in}}{d_{out}+d_{in}}$).

The modified Wheeler approximation formula is derived from the Wheeler’s formulas. In [36], Wheeler presented several formulas for the calculation of the inductance of helical coils with one or more layers, and Mohan et al. proposed an approximation to make Wheeler’s formulas valid for planar coils [35]. This way, they derived an equation that is dependent on two coefficients ($k_{1}$ and $k_{2}$) related to the coil layout, the vacuum permeability, and some geometric parameters of the coil such as the number of turns (*N*), its average diameter ($d_{avg}$), and fill ratio (ρ). Table 3 shows the valid coil layouts and the values of the corresponding coefficients to apply in:

(3) $$L=k_{1}\mu_{0}\frac{N^{2}d_{avg}}{1+k_{2}\rho }.$$

Since this approximation formula depends only on the average diameter and the fill ratio of the coil, it is not sensible to different configurations of *s* and *w* when the values of $d_{avg}$ and ρ are the same. In these cases, the error associated with this model increases.

The popularity of these three approximation formulas is quite distinct. From the research works found in the literature, it is possible to state that the Current Sheet Approximation formula is the most used [8,19,20,21,22,23,24,37,38], followed by the Modified Wheeler [24,30] and the Data Fitted Monomial [26,39].

Regarding the calculation of the inductance of a multi-layer planar coil, it depends on the parameterization of the coil at each layer (geometry, number of turns, space between turns, wire width, internal and external diameter) and the distance between them. Thus, to obtain the total inductance of a 2-layer coil it is necessary to sum the self-inductances of each layer, and to sum or subtract, depending on the direction of the current, the double of the mutual inductance between the layers [20,21,33]:

(4) $$L_{T}=L_{1}+L_{2}\pm 2M$$

where $L_{T}$ is the total inductance, $L_{1}$ and $L_{2}$ are the values of self-inductance of the coils in each layer, and *M* is the mutual inductance between the two coils, that can be calculated through:

(5) $$M=K\sqrt{L_{1}L_{2}}.$$

where *K* is the coupling coefficient between the coils of each layer. Currently, to perform these calculations, the combination of the coupling coefficient with the self-inductance values obtained by the previously mentioned approximation formulas is being used [37,40]. Typically, this coefficient is either calculated analytically [24] or using a numerical model [25], or it can even be measured experimentally [41]. Equation (6) is the analytical expression that is commonly used to calculate the coupling coefficient between coils [20,21,24]. It was derived experimentally by Zhao through multi-layer coils with the distance between adjacent layers varying from 0.75 mm to 2 mm, considering coils with 5 to 20 turns [20,21,24]. This equation has the particularity of being valid only within the range that was derived, and the coils of all layers must be equal and perfectly aligned.

(6) $$\begin{array}{c} M=2K_{c}\sqrt{L_{1}L_{2}}\\ K_{c}=\frac{N^{2}}{0.64[\left(0.184X^{3}-0.525X^{2}+1.038X+1.001\right)\left(1.67N^{2}-5.84N+65\right)]} \end{array}$$

where, $K_{c}$ is the coupling factor, *N* the number of coil’s turns, and *X* the distance between the layers in mm [20,21,24].

In short, it can be concluded that defining the design of a 1-layer planar coil using one of the three approximation formulas is a simple, fast process, with no need for high computational resources. However, as previously stated, these formulas are only valid for certain coil geometries and do not have the same accuracy as a FEM simulation. It can even be said that its accuracy is affected by the geometric configuration of the coil under analysis, for example, in the case of the Data Fitted Monomial expression, its errors will be greater for situations in which the coils are outside the equation deduction ranger. In the case of Current Sheet Approximation, the greater the space between turns, in relation to the width of the wire, the greater the errors; and in the case of the Modified Wheeler coils with different combinations of space between turns and the width of the track, but with the sum of the two constant, it will result in the same inductance result. When considering 2-layer planar coils, one of the three equations referred before can be used together with an auxiliary equation to determine the coupling coefficient between layers. The simplicity, speed, and low cost of the analytical inductance calculation process, for 1-layer planar coils, can, most of the time, be achieved by using these models found in the literature. However, the limitation of the coupling coefficient equation itself, which is only valid for coils with 5 to 20 turns and with adjacent layers spaced between 0.75 mm and 2 mm, does not allow these solutions to be considered viable for all configurations of multi-layer planar coil calculation.

Taking into account the limitations of the analytical methods presented, the constraints of the FEM simulations, and the growing interest in products with multi-layer planar coils, it becomes desirable for several industries to have a versatile, fast, open source, and accurate tool to speed up the design process. In this paper an approximation model is proposed, combining versatility in the coil layout, with speed, accuracy, easy management and interaction.The following section describes the proposed tool that, unlike the other methods, matches the needs of the industries.

## 3. Methods: Analytical Model for Planar Coil Inductance Calculation

The main goal of the proposed model is to be able to calculate a coil self-inductance, regardless of its geometry or number of layers. The coil is treated as a group of connected segments, where its inductance is calculated through the sum of the self-inductance of every conductor segment, plus the mutual inductance between each of the segments, using Grover equations [33,42]. These equations are considered by the literature [8,30,31,37,43] as the most accurate, but as there is no model developed from them to be used directly on coils of arbitrary geometry, they have not been used.

**Figure 2:**
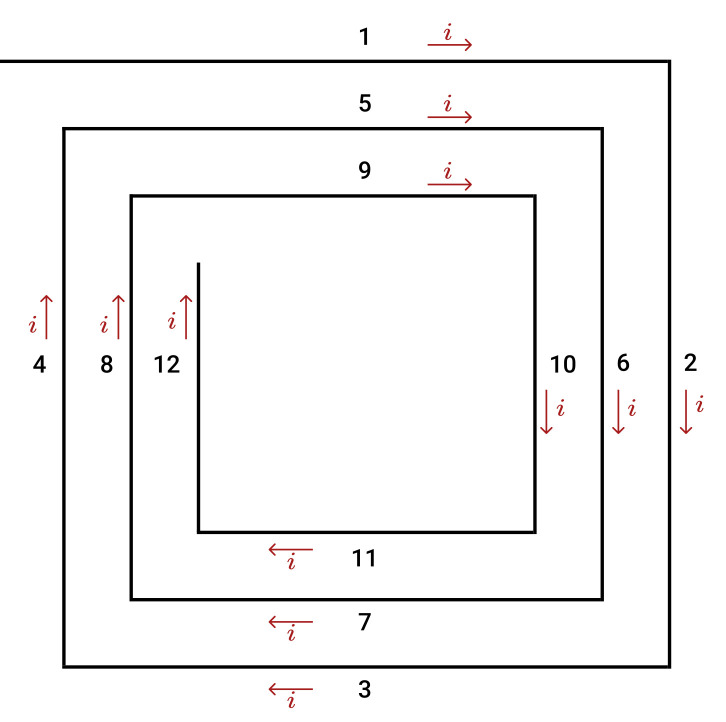
Square planar coil with three turns.

**Figure 3:**
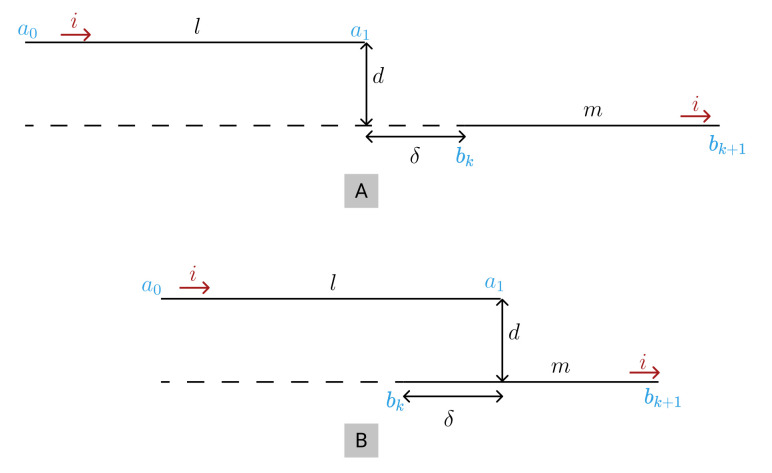
Mutual Inductance’s general case of two parallel filaments, (**A**): two filaments non overlapped; (**B**): two filaments overlapped.

Considering the coil’s geometry example of Figure 2, its inductance, $L_{T}$, can be calculated using:

(7) $$\begin{array}{c} L_{T}=L_{0}+M_{+}-M_{-}\\ L_{0}=L_{self1}+L_{self2}+L_{self3}+L_{self4}+L_{self5}\\ +L_{self6}+L_{self7}+L_{self8}+L_{self9}\\ +L_{self10}+L_{self11}+L_{self12}\\ M_{+}=2(M_{1,5}+M_{2,6}+M_{3,7}+M_{4,8}+\\ +M_{5,9}+M_{6,10}+M_{7,11}+M_{8,12})\\ M_{-}=2(M_{1,7}+M_{1,3}+M_{1,11}+M_{5,7}+M_{5,3}+M_{5,11}\\ +M_{9,7}+M_{9,3}+M_{9,11}+M_{2,8}+M_{2,4}+M_{2,12}\\ +M_{6,8}+M_{6,4}+M_{6,12}+M_{10,8}+M_{10,4}+M_{10,12}) \end{array}$$

where $L_{T}$ is the total inductance of the coil; $L_{selfi}$ is the self-inductance of straight conductor *i*; $M_{+}$ is the mutual inductance of segments with currents in the same direction; and $M_{-}$ is the total mutual inductance of segments with currents in opposite directions. The self-inductance of a conductor with a rectangular cross-section can be determined by:

(8) $$L_{self}=0.002l\left(\ln\left(\frac{2l}{w+t}\right)+0.50049+\frac{w+t}{3l}\right)$$

where *l* (cm) is the conductor length; *w* (cm) the wire width; and *t* (cm) its thickness. The mutual inductance between parallel segments (Figure 3) can be calculated using [42]:

(9) $$M=\frac{\mu_{0}}{4\times \pi }\left[\sigma \sinh^{-1}\frac{\sigma }{d}-\zeta \sinh^{-1}\frac{\zeta }{d}-\gamma \sinh^{-1}\frac{\gamma }{d}+\delta \sinh^{-1}\frac{\delta }{d}-\sqrt{\sigma^{2}+d^{2}}+\sqrt{\zeta^{2}+d^{2}}+\sqrt{\gamma^{2}+d^{2}}-\sqrt{\delta^{2}+d^{2}}\right]$$

where σ=l+m+δ, ζ=l+δ, and γ=m+δ, being *l* and *m* the length of the segments visible in Figure 3 in cm. In case the segments overlap (Figure 3B), it becomes σ=l+m−δ, ζ=l−δ, and γ=m−δ.

**Figure 4:**
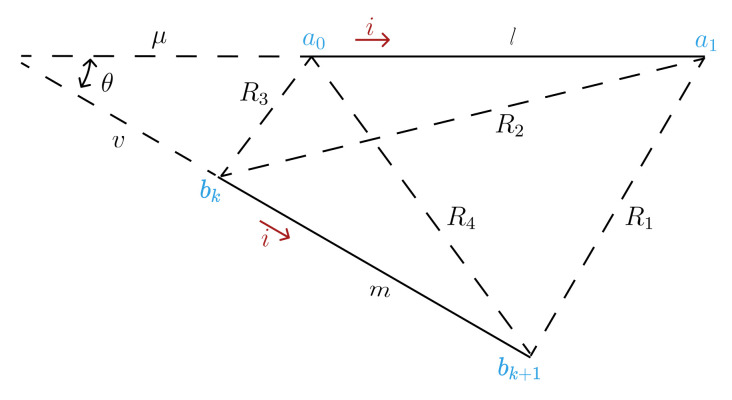
Mutual Inductance’s general case of two nonparallel filaments.

For two nonparallel segments their mutual inductance can be determined by [42]:

(10) $$M=\frac{\mu_{0}}{2\times \pi }\cos\theta \left[\left(\mu +l\right)\tanh^{-1}\frac{m}{R_{1}+R_{2}}+\left(v+m\right)\tanh^{-1}\frac{l}{R_{1}+R_{4}}-\mu \tanh^{-1}\frac{m}{R_{3}+R_{4}}-v\tanh^{-1}\frac{l}{R_{2}+R_{3}}\right]$$

(11) $$\begin{array}{c} 2\cos\theta =\frac{\alpha^{2}}{lm}\alpha^{2}={R_{4}}^{2}-{R_{3}}^{2}+{R_{2}}^{2}-{R_{1}}^{2} \end{array}$$

(12) $$\begin{array}{c} \mu =\frac{[2m^{2}\left({R_{2}}^{2}-{R_{3}}^{2}-l^{2}\right)+\alpha^{2}\left({R_{4}}^{2}-{R_{3}}^{2}-m^{2}\right)]l}{4l^{2}m^{2}-\alpha^{4}} \end{array}$$

(13) $$\begin{array}{c} v=\frac{[2l^{2}\left({R_{4}}^{2}-R_{3}^{2}-m^{2}\right)+\alpha^{2}\left({R_{2}}^{2}-{R_{3}}^{2}-l^{2}\right)]m}{4l^{2}m^{2}-\alpha^{4}} \end{array}$$

(14) $$\begin{array}{c} {R_{1}}^{2}={\left(\mu +l\right)}^{2}+{\left(v+m\right)}^{2}-2\left(\mu +l\right)\left(v+m\right)\cos\theta \\ {R_{2}}^{2}={\left(\mu +l\right)}^{2}+v^{2}-2v\left(\mu +l\right)\cos\theta \\ {R_{3}}^{2}=\mu^{2}+v^{2}-2\mu v\cos\theta \\ {R_{4}}^{2}=\mu^{2}+{\left(v+m\right)}^{2}-2\mu \left(v+m\right)\cos\theta \end{array}$$

with l,m representing the length of the segments in cm, θ the angle between segments, and $R_{1}$, $R_{2}$, $R_{3}$, and $R_{4}$ the distance between their terminals (Figure 4).

## 4. Results and Discussions

The proposed model was validated by comparing the results with well-known analytical formulas mentioned in Section 2 (for the geometries to which they are applicable), considering single and multi-layer planar coils [20], as well as with FEM simulations and experimental measurements made on PCB printed coils. Apart from the different number of layers, other coil’s parameters have been tested during validation, such as line width (*w*), space between turns (*s*), number of turns (*N*), and number of segments per turn ($N_{s}$). As the typical analytical formulas are valid just for coils with 4, 6, and 8 segments per turn, the coils with 10 and 12 segments in each turn were validated only using FEM results and the experimental measurements. In what concerns *w* and *s*, considering the limitations of the Current Sheet Approximation formula, the same values were considered for both parameters (s=w), specifically 0.15 mm and 0.10 mm. It was also taken into account that, according to [20], the coupling factor expression required for multi-layer calculations is only valid for coils with 5 to 20 turns with a distance between layers from 0.75 mm to 2.0 mm. All the settings used in the different simulation cases are summarized in Table 4.

### 4.1. Analytical Models vs. FEM Model

In this section, a comparison between the coil’s inductance values obtained with the proposed model, with the analytical formulas, and with the FEM simulations, used as the reference values, was performed.

**Figure 5:**
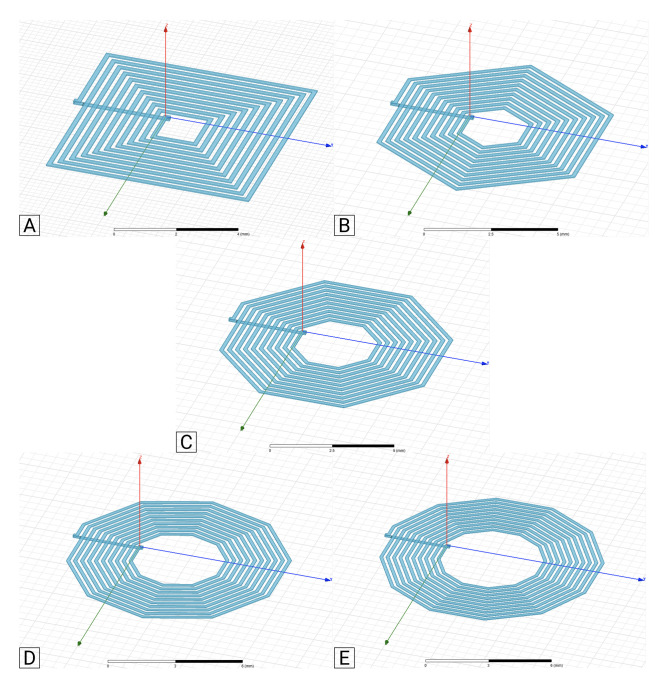
1-layer coil layouts simulated in Ansys Software. Segments per turn: (**A**): 4; (**B**): 6; (**C**): 8; (**D**): 10; (**E**): 12.

**Figure 6:**
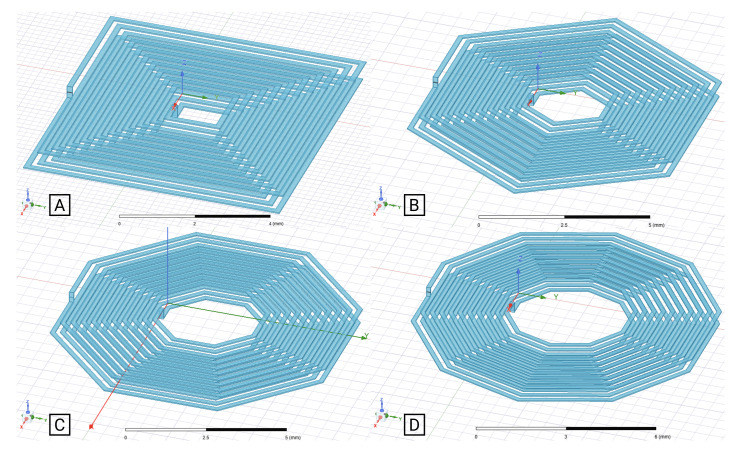
2-layer coil layouts simulated in Ansys Software. Segments per turn: (**A**): 4; (**B**): 6; (**C**): 8; (**D**): 10.

Figure 5A–E and Figure 6 show the example geometries for coils with 1- and 2-layer, respectively, used in this analysis. As mentioned before, due to the applicability limitations of the traditional analytical formulas, they were only applied to layouts A–C from Figure 5 and Figure 6.

**Figure 7:**
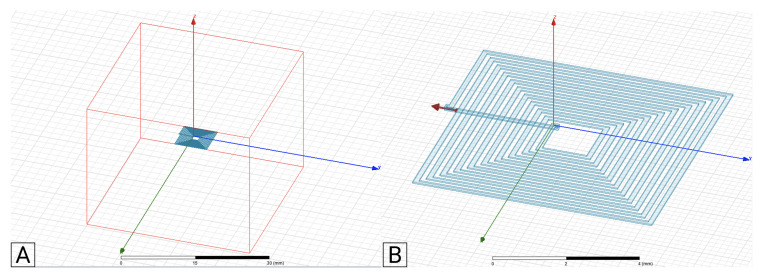
(**A**): Ansys project with 1-layer coil and air box. (**B**): Current applied into the coil.

**Figure 8:**
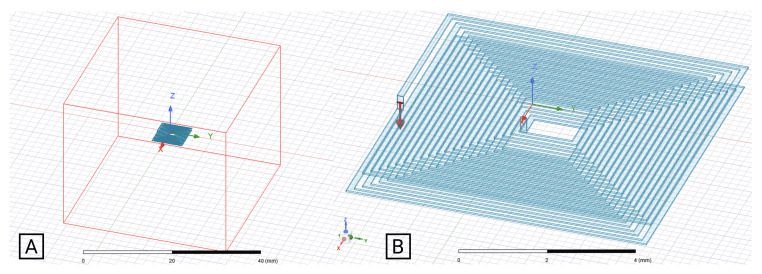
(**A**): Ansys project with 2-layer coil and air box. (**B**): Current applied into the coil.

The FEM simulations were performed in Ansys Electronic Software as a magnetic problem, specifically the Eddy current mode. It was also studied as a magnetostatic problem, but was not considered to be relevant for this study, considering the experimental validation to be performed. The FEM model considers an air-box with a side dimension of five times the coil outer diameter. Previous studies ensure that bigger air-box dimensions do not change the simulation results in more than 1%, as shown in Figure 7A for a 1-layer square coil example, and Figure 8 for a 2-layer square coil example. A test current of 1 mA was used in both models, with the solver frequency selected to be 1 MHz, in order to match the experimental test frequency. The adaptive setup was configured with a percent error of 1%, and a minimum of two convergence steps.

**Figure 9:**
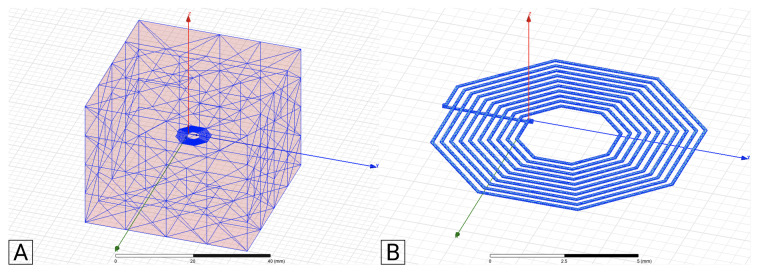
(**A**): Air box’s mesh. (**B**): Coil’s mesh.

Regarding the mesh parameters, the type selected for the simulation cases under analysis was the surface approximation based, for both the coil and the air-box. Around the coil and the coil itself, it was assigned a finer mesh (e.g., Figure 9B), since this was the critical area for the calculation of self-inductance, while for the air-box a coarser mesh was used (e.g., Figure 9A).

**Figure 10:**
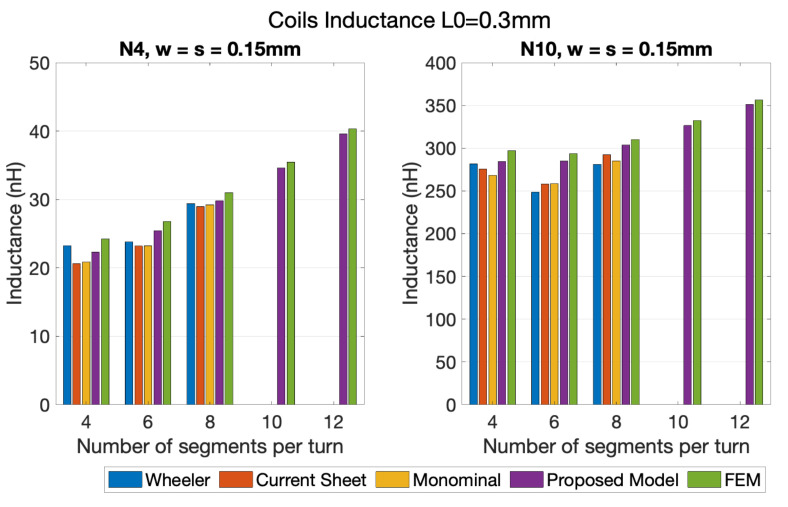
Comparison between several methods of inductance calculation of coils with 4 and 10 turns, for $L_{0}=$ 0.3 mm, w=s= 0.10 mm, and for 4, 6, 8, 10, and 12 segments per turn.

**Figure 11:**
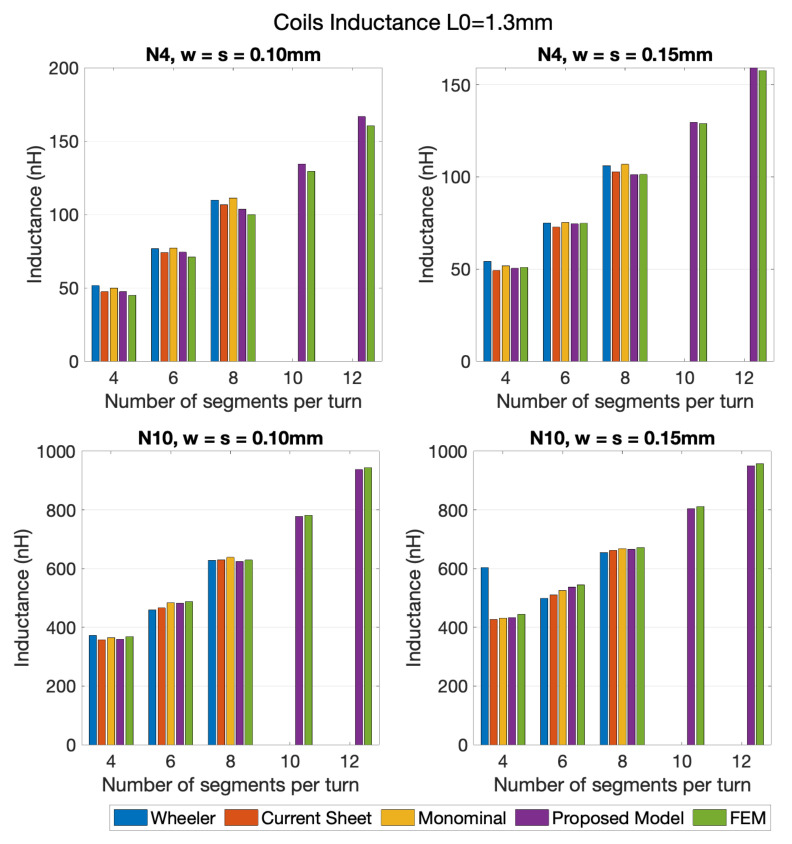
Comparison between several methods of inductance calculation of coils with 8 and 10 turns, for $L_{0}=1.3$ mm, w=s= 0.10 mm and w=s= 0.15 mm, and for 4, 6, 8, 10, and 12 segments per turn.

In Figure 10 and Figure 11, the self-inductance results for the different 1-layer coil layouts (see Figure 5A–E) calculated using the different models are shown. Figure 10 presents inductance values for coils with 4 and 10 turns, with an inner segment of 0.3 mm, and space between turns and wire’s width of 0.15 mm.

Considering the FEM results as the reference, the set of results shown in Figure 10 show that the range of errors in results from the Modified Wheeler method ranged from 0.99% to 12.67%, and from 2.93% to 9.39%, using the Current Sheet Approximation method. The error registered with the Data Fitted Monomial was from 2.08% to 9.31%, and from 2.13% to 7.93%, using the proposed model. In the remaining coil variations analyzed (coils with 10 and 12 segments per turn), the proposed model shows errors between 1.39% and 2.45%. Figure 11 details the data from coils with 4 and 10 turns, with an inner segment of 1.3 mm, and with two combinations of the space between turns (*s*) and the wire’s width *w*, w=s=0.10 mm and w=s=0.15 mm. Through querying the graphs it is possible to verify that the Modified Wheeler method, for N4 had an error range of [0.35–15.04%], and for N10 of [0.18–35.85%]. Applying a similar analysis, the Current Sheet Approximation method had an error range of [1.45–6.74%] for N4 and [0.04–6.31%] for N10, while the Data Fitted Monomial showed [0.69–11.31%] for N4 and [0.55–3.51%] for N10. Regarding the model proposed in this work, for the four turns coils the errors obtained were between 0.06% and 5.90%, and for 10 turns between 0.78% and 2.47%. Particularly, if the 10 and 12 segment coil geometries were considered, the error range dropped to values between 0.59% and 3.99% for N4, and 0.62% and 0.79% for N10. Given that, it is possible to see that the errors of the proposed model were at the same level as the error range from the generic formulas, with the significant advantage of not showing any geometry related limitation, and presenting lower error rangers than some of the approximated expressions.

**Figure 12:**
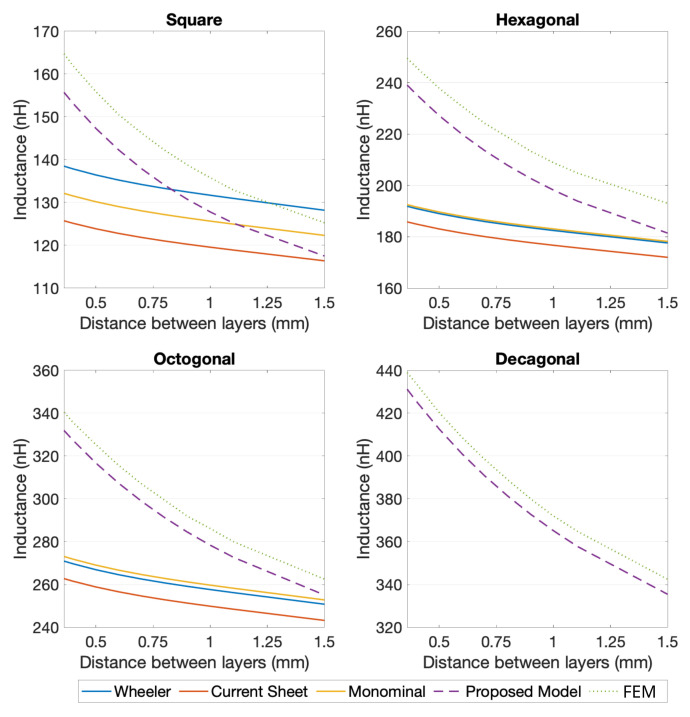
Comparison between several methods of inductance calculation of 2-layer coils with 4 turns, for $L_{0}=$ 1.3 mm, w=s= 0.15 mm, and for 4, 6, 8, and 10 segments per turn.

**Figure 13:**
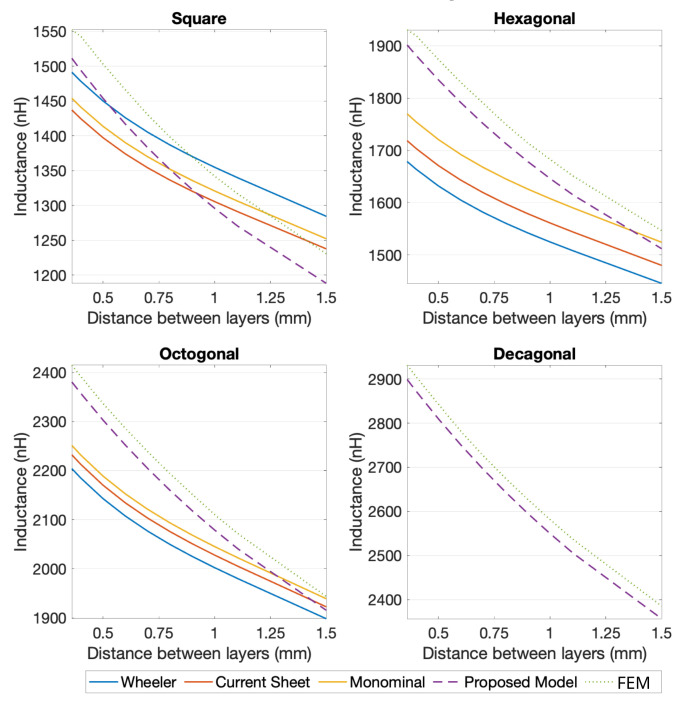
Comparison between several methods of inductance calculation of 2-layer coils with 10 turns, for $L_{0}=$ 1.3 mm, w=s= 0.15 mm, and for 4, 6, 8, and 10 segments per turn.

The same validation procedure was used for the two-layer planar coils. Considering that, and in order be able to calculate the self-inductance of multi-layer coils using the generic expressions, an additional calculation had to be performed (for the coupling coefficient between the different layers) [20,21]. Based on that, and analyzing the results from Figure 12 and Figure 13, it is noticeable that the proposed model retrieved a more accurate calculation, when compared to the generic expressions. In view of the results obtained for the 10 turns two-layer coils, Figure 13, the generic expression showed a better performance than for the four turns two-layer ones, Figure 12, with errors as high as 4.42% for the square geometry, 10.83% for the hexagonal and 6.57% for the octagonal, if the distance between coils was limited to a range of 0.75 mm to 2 mm (limitation of the coupling coefficient calculation for the generic expressions). For the same selected cases, the proposed model showed maximum errors of 3.53% for the square coils, 2.22% for the hexagonal coils, 1.56% for the octagonal coils, and 1.23% for the decagonal coils.

It can be concluded that, even within the range in which the generic expressions coupling coefficient calculation can be applied, the results obtained using the proposed model were more accurate, with small differences to the outcome of the FEM simulation. Additionally, if the strict geometry limitations of the generic expressions and the coupling coefficient calculation were taken into consideration, it can easily be stated that the developed model was capable of joining reliable results to a versatile calculation method.

### 4.2. Experimental Validation

**Figure 14:**
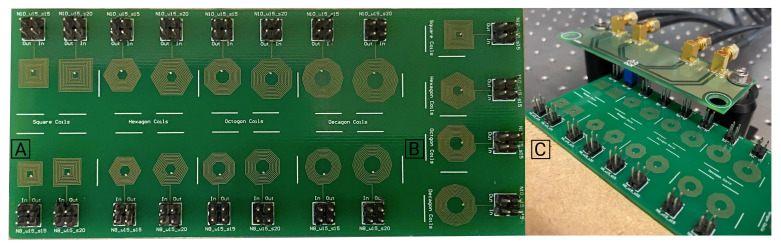
The PCB produced has coils with 4, 6, 8, and 10 segments per turn, all with an inner segment of 1.3 mm. In A: are represented the one-layer coils, in the top line are coils with ten turns and in the bottom line coils with eight turns; in B: are the two-layer coils, with ten turns; and in C: the measurement setup.

In order to have a complete validation of the proposed model, experimental measurements of self-inductance were performed of several manufactured coil geometries. For this experiment, the minimum line width (*w*) and spacing (*s*) used in the test coils were of 0.15 mm, due to PCB manufacturer limitations. Figure 14A shows the PCB produced with the different one-layer coils namely 4, 6, 8, and 10 segments per turn, with an inner segment of 1.3 mm. These coils were grouped in two sets, one with ten turns (on the top line) and another with eight turns (on the bottom line).

**Figure 15:**
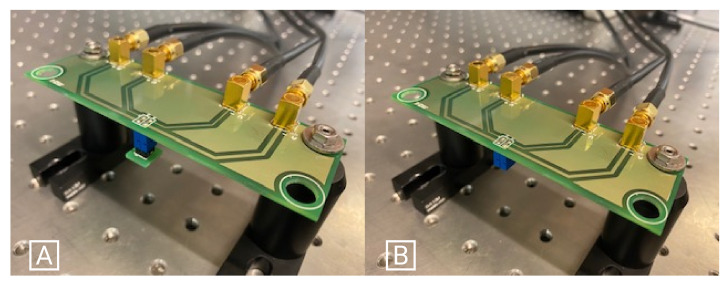
Calibration setup. (**A**): Short-circuit. (**B**): Open-circuit.

In each line and for each geometry, there was a pair of coils, one with w=s= 0.15 mm, and another one with w= 0.15 mm and s= 0.20 mm. In Figure 14B the two-layer coils were visible, showing the different geometries of 4, 6, 8, and 10 segments per turn, with an inner segment of 1.3 mm. For the two-layer coil, only coils with 10 turns were fabricated. The distance between each coil was chosen in a way that the space was maximized, without creating any effect in the single coil experimental measurement. This was confirmed by comparing the results of a single coil board with the multi-coil measurement setup, without detecting major differences. To minimize any error in the measurement setup, as well as in the coil manufacturing process, two sets of PCBs were produced and the self-inductances in both of them were measured thirteen times. The Keysight Technologies E4980AL-102 LCR precision meter, was used for the inductance measurements at the test frequency of 1 MHz, with a four point Kelvin customized probe set-up. In order to minimize measurement errors, the measurement setup depicted in Figure 14B was developed to plug the coils’ PCB to the LCR meter, after the calibration process (Figure 15).

**Figure 16:**
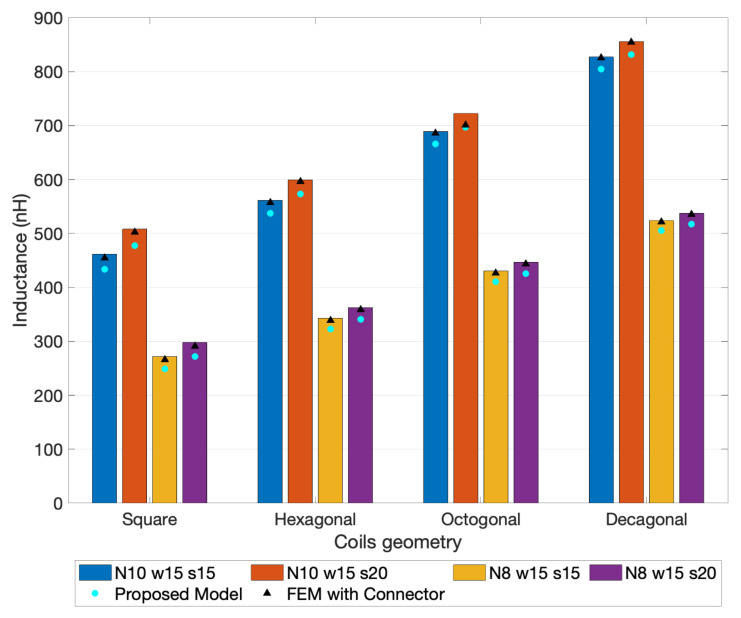
Graphic with the average inductance values from the experimental measurements and FEM simulation results.

**Figure 17:**
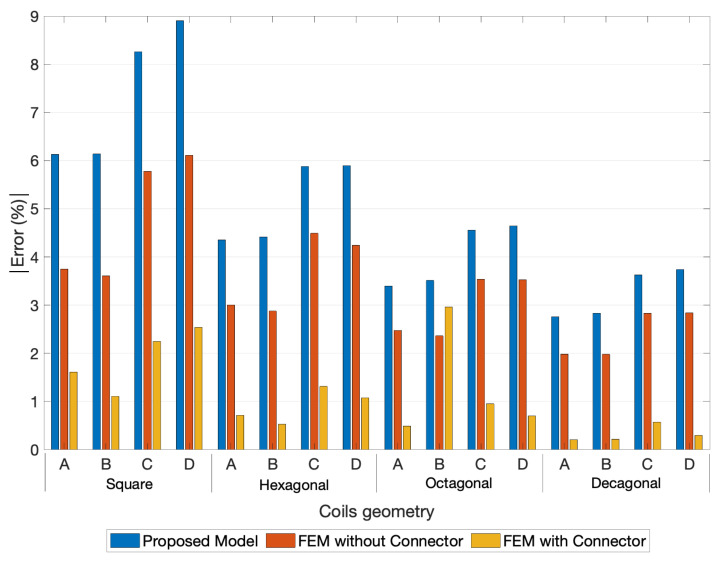
Relative errors module between the Experimental Measurements, the FEM simulations and the proposed model for the 1-layer coils. A-[N=10, w= 0.15 mm, and s= 0.15 mm]; B-[N=10, w= 0.15 mm, and s= 0.20 mm]; C-[N=8, w= 0.15 mm, and s= 0.15 mm]; D-[N=8, w= 0.15 mm, and s= 0.20 mm].

**Figure 18:**
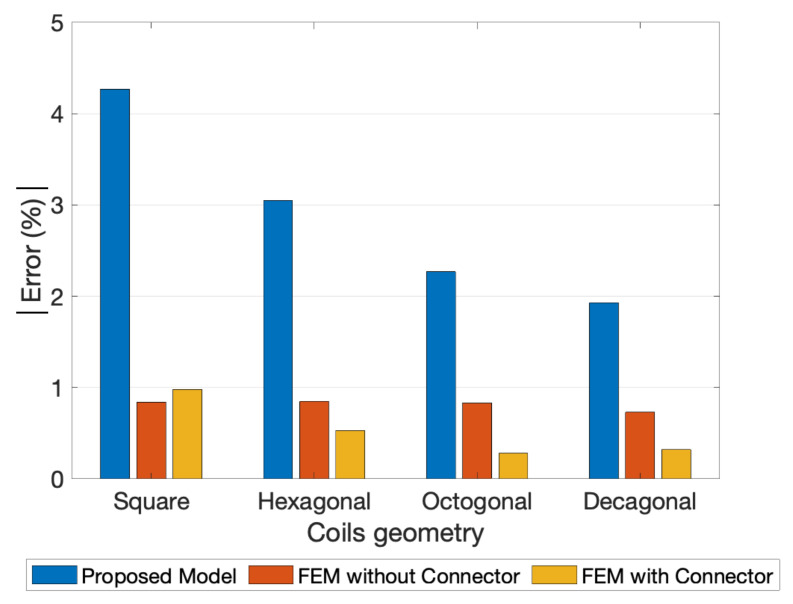
Relative errors module between the Experimental Measurements, the FEM simulations and the proposed model for the 2-layer coils with 10 turns, w=s= 0.15 mm, and a 1.3 mm inner segment.

To compare the experimental measurement of the fabricated coils with the FEM model used as reference, the thirteen LCR measurements were averaged. During the experimental evaluation process, the maximum deviation registered in both PCBs was around 0.68% for the one-layer coils, and 0.48% for the 2-layer coils, which proves the high precision of the measurements. The experimental, FEM simulations with connector, and the proposed model results for the one-layer coil designs are depicted in Figure 16. The detailed analyses of the relative errors between the experimental measurements are visible in Figure 17 and Figure 18.

In Figure 14A, it is noticeable that to perform the measurements, two traces were added to the printed coils, from the coil’s extremities to the connectors. For this reason, it was expected that the inductance values measured with the LCR should be higher than the ones obtained from the model, and the FEM simulations presented before (as it only considered the coil element). In order to understand the impact and quantify this effect, a FEM model was made with two connecting traces coming out the coil towards the air box.

As predicted, when considering the connecting traces, higher inductance was registered and the error to the experimental measurement was minimized. Comparing the data for single layer coil’s from FEM model, one can observe that considering the connecting wires led to an error reduction of around 2% for N=10, and 3% for N=8.

Finally, the results calculated by the proposed model were compared with experimental measurements, as presented in Figure 17 and Figure 18. The errors of the FEM models, with and without connectors, were also added to the representation always using the experimental measurement as reference. Note that the coils layouts simulated with the proposed model did not include the extended connecting traces (see Figure 5 and Figure 6).

Comparing the errors of the FEM model without connectors and the ones from the proposed model, it is perceptible that the major contribution to these errors was from the absence of the connecting traces in the analysis. Even with this geometry difference, the errors between the experimental measurements and the ones from the proposed model remain at an acceptable range, being in the case of the one-layer coils smaller than 9% for square coils, 6% for hexagonal coils, 5% for octagonal coils, and 4% for decagonal coils. In the case of two-layer coils, it was further minimized to values smaller than 5% for square coils, 3% for hexagonal coils, 2.5% for octagonal coils, and 2% for decagonal coils. It can thus be concluded that the proposed model was valid and has good accuracy for both one- and two-layer coils induction calculation.

## 5. Conclusions

A versatile, fast, and comprehensive tool to estimate the self-inductance of planar coils is proposed and validated in this paper. The model was validated by comparison with generic analytical expressions, FEM simulations, and experimental measurements on manufactured one- and two-layer coils. The results of the model for the different coil geometries show errors, when compared to experimental measurements, always below 10% in the case of the one-layer coils, and below 5% in the two-layer coils. In the case of the one-layer coils, these errors can be considerably decreased if the coil’s layout used in the analytical analysis with the proposed model considered the filaments of the connections to the measurement connectors, as in the fabricated PCB based coils experimentally used. This tool can be further explored to calculate in a fast and reliable way the coupling coefficient between coils, without any geometry or distance limitation, as commonly verified in other calculation methods. 

## Figures and Tables

**Table 1:** Coefficients for Data Fitted Monomial formula.

Layout	β ${}^{*}$	$\alpha_{1}$	$\alpha_{2}$	$\alpha_{3}$	$\alpha_{4}$	$\alpha_{5}$
Square	1.62	−1.21	−0.147	2.40	1.78	−0.030
Hexagonal	1.28	−1.24	−0.174	2.47	1.77	−0.049
Octagonal	1.33	−1.21	−0.163	2.43	1.75	−0.049

* Values $\times 10^{-3}$.

**Table 2:** Coefficients for Current Sheet Approximation Formula.

Layout	$c_{1}$	$c_{2}$	$c_{3}$	$c_{4}$
Square	1.27	2.07	0.18	0.13
Hexagonal	1.09	2.23	0.00	0.17
Octagonal	1.07	2.29	0.00	0.19
Circle	1.00	2.46	0.00	0.20

**Table 3:** Coefficients for Modified Wheeler formula.

Layout	$k_{1}$	$k_{2}$
Square	2.34	2.75
Hexagonal	2.33	3.82
Octagonal	2.25	3.55

**Table 4:** Summary of the different simulation groups.

Number of Layers	$L_{0}$${}^{*}$(mm)	w=s (mm)	*N*
1	0.3	0.15	4
			10
	1.3	0.10	8
		0.15	10
2	1.3	0.15	10

* $L_{0}$: inner segment’s length, *w*: wire’s with, *s*: space between turns, *N*: number of turns.

## Data Availability

Not applicable.
